# Interfacial Characterization of the Electrochemical Adsorption of Caffeine on Poly(pyrrole) Nanotubes/Silica

**DOI:** 10.1021/acsomega.5c10006

**Published:** 2025-11-21

**Authors:** Tatiana Lima Valerio, Camilla K. Boaron, Luis F. Marchesi, Bruno José G. da Silva, Marcio Vidotti

**Affiliations:** † Grupo de Pesquisa Em Macromoléculas e Interfaces (GPMIn), Departamento de Química, 28122 Universidade Federal Do Paraná (UFPR), CP 19032, 81531-980 Curitiba, Paraná, Brazil; ‡ Grupo de Estudos em Espectroscopia de Impedância Eletroquímica (GEIS), Universidade Tecnológica Federal Do Parana, Rua Dr.Washington Subtil Chueire, 330. Jd. Carvalho, CEP 84017-220 Ponta Grossa, Paraná, Brazil; § Grupo de Cromatografia e Técnicas de Microextração (CROME)—Departamento de Química, Universidade Federal do Paraná, C.P. 19032, 81531-980 Curitiba, Paraná, Brazil

## Abstract

In this work, a polypyrrole nanotube/silica (PPyNT/SiO_2_)-modified electrode was prepared by an all-electrochemical route and characterized by electrochemical, spectroscopic, and microscopy analyses; scanning electron microscopy confirmed the superimposition of a particulate silica on the PPyNTs without any loss of electroactivity of conducting polypyrrole. The PPyNTs/SiO_2_ electrode was employed for the electroadsorption of caffeine, where it was found that in its less oxidized form, the PPyNTs boosted the adsorption capability of intrinsic silica. Electrochemical impedance spectroscopy (EIS) modeling was employed and modeled with equivalent circuit methodology, and all the results were compared with the Sips isotherm model, showing that the PPyNTs/SiO_2_ electrode presented a more heterogeneous surface (*n*_S_ = 1.25) and a nearly 2-fold increase in maximum adsorption capacity (*q*_ms_) compared to pristine PPyNTs.

## Graphical abstract

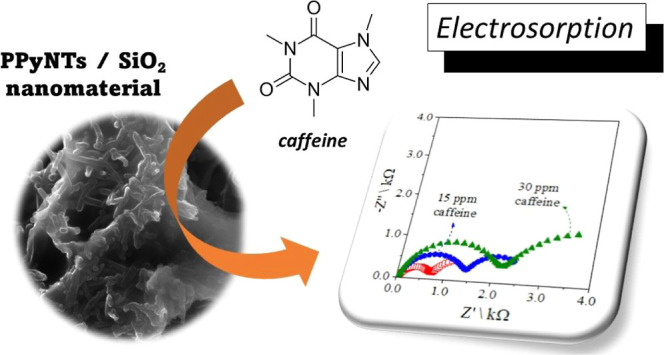

## 1 Introduction

The presence of emerging pollutants, such as synthetic chemicals, pesticides, pharmaceuticals, and personal care products in industrial, agricultural, and municipal wastewater, represents a global problem for human health and the aquatic ecosystem, both in developed and developing countries.^1^ Recent studies suggest that many of these pollutants can cause adverse effects on human health and aquatic ecosystems.^1–4^ In addition, they have the potential to act as endocrine system disruptors, affecting the natural functioning of this system in humans and animals.^2^ Among them is caffeine, commonly found in drinking water, groundwater, wastewater, and effluents from wastewater treatment plants, rivers, lakes, seas, and even Antarctic waters.^5,6^ One of the best indicators of anthropogenic action in the aquatic environment is the presence of caffeine, which has a short half-life.^7^ If it is present, it indicates that there has been recent contamination from domestic sewage, indicating that the aqueous matrix is polluted and that there is a high probability of potentially dangerous contaminants and other pharmaceutical compounds also being present.^8–10^

The lack of efficient methods to remove emerging pollutants makes remediation of their negative effects on aquatic ecosystems and human health even more difficult.^11–13^ Adsorption is a common technology for removing organic pollutants from wastewater, achieving high levels of adsorption, as observed by Portinho et al.,^14^ who obtained the maximum adsorption capacity of 395 mg g^–1^ using grape skin activated carbon. But the regeneration step generally requires the use of chemicals, such as additional organic solvents that end up causing secondary pollution; for example, Danish et al.^15^ employed activated carbon from *Acacia mangium* wood to adsorb caffeine, and desorption was carried out with a solution of 95% ethanol. In this sense, adsorption with electrochemical control or electrosorption offers an attractive solution with the application of a specific potential in the adsorption and desorption of emerging pollutants.^12,16,17^ Electrosorption is a process that employs an external electric potential to control the interaction between a pollutant and the surface of an electrode. Unlike conventional adsorption, where the driving force is the natural affinity between the adsorbate and the adsorbent, in electrosorption, the attractive force is modulated by an electric field. It can occur by an electric double layer (EDL) mechanism, which is the main mechanism for ion removal.^18–20^ The application of potential generates an electric double layer at the electrode–electrolyte interface. In the case of neutral molecules such as caffeine, the polarized electrode surface can induce a dipole in the molecule, resulting in an electrostatic attraction that facilitates adsorption.^19,21^

Electrode materials in a system for electrochemically mediated adsorption need to exhibit three properties: (1) electrical conductivity to respond to applied potentials, (2) different affinities with organic species depending on the applied electrochemical modulation, and (3) a high surface area for interactions with organic species to promote a high adsorption capacity.^13^ Currently, the preparation of composite electrode materials with carbon materials, metal oxide materials, and conductive polymeric materials has become a focus of research.^11,12,22^ Among the conductive polymers, polypyrrole (PPy) stands out for its high specific capacitance, low cost, and good chemical stability, in addition to the possibility of modulating selectivity for different molecules through its doping and the application of an electrical potential.^11,22^ This characteristic is particularly interesting: the applied potential not only alters the surface charge but can also modulate specific molecular interactions. For example, the potential can weaken or strengthen van der Waals interactions, London forces, or π–π stacking interactions between the electrode surface and the contaminant molecule, which has aromatic rings. This is a more subtle but crucial form of electrochemical control.^23,24^ The combination of PPy with SiO_2_ is attractive when the aim is to improve the adsorption capacity, mainly due to the high specific surface area of silica and highly porous structures,^25^ providing the PPy/SiO_2_ composite the main characteristics mentioned above as necessary for an electrode for electrochemical adsorption systems.^26,27^ In this work, aiming to obtain a high electrosorption capacity, a PPy/SiO_2_ composite was obtained using only electrochemical routes for the synthesis of both materials, making the obtaining process simpler and faster. The obtained compound was used as a working electrode in the electrochemically controlled adsorption of caffeine, which was used as a model molecule.

## 2 Materials and Methods

### 2.1 Materials

All solutions were prepared by using ultrapure water (*R* = 18.2 MΩ cm^2^, ElgaLab system). The following reagents used were of analytical grade: pyrrole (98%), tetraethyl orthosilicate (TEOS), methyl orange (MO), ethylenediaminetetraacetic acid (EDTA), potassium nitrate (KNO_3_), caffeine 99% (Sigma-Aldrich), and potassium chloride (KCl), which were purchased from Sigma-Aldrich Chemical (St. Louis, MO, USA), and nitric acid (HNO_3_), which was purchased from Synth (Diadema, SP, Brazil). The monomer pyrrole was distilled under low pressure, bubbled with N_2_, and kept in the freezer until its use.

### 2.2 Synthesis of PPyNTs/SiO_2_

The electrochemical procedures were performed in an Autolab PGSTAT204 potentiostat. 316 stainless steel 400 mesh (1 × 3.5 cm) was used as a working electrode for the deposition of poly­(pyrrole) nanotubes (PPyNTs) and SiO_2_ over poly­(pyrrole) nanotubes (PPyNTs/SiO_2_). The counter electrode (CE) used was platinum, and the reference electrode (RE) was Ag/AgCl (sat). The deposition of PPyNTs followed the methodology already well established in Hryniewicz et al.,^28^ where the deposition was made on the electrode in an area of 0.5 cm^2^ and was used in a solution containing 50 mmol L^–1^ pyrrole, 8 mmol L^–1^ KNO_3_, and 5 mmol L^–1^ of MO. The pH was adjusted to 2 with HNO_3_. The synthesis was performed by applying 0.8 V with a charge control of 500 mC cm^–2^. After deposition, the PPyNT electrode was removed from the cell, washed with water and 70% ethanol, and dried in an oven at 30 °C.

For the subsequent deposition of silica, this PPyNT electrode was used as a WE, using an adapted methodology based on the methodology of Salinas-Torres et al. and Walcarius.^29,30^ Applying a potential of −1.2 V with charge control of 150 mC cm^–2^ in a solution containing 4.5 mol L^–1^ TEOS, 17 mol L^–1^ ethanol 99.98%, and 0.1 mol L^–1^ KCl, the pH of the solution was adjusted to 3 with HCl 0.1 mol L^–1^. The resulting PPyNTs/SiO_2_ composite electrode was rinsed with deionized water and ethanol and dried at room temperature before further characterization.

### 2.3 Characterization

Scanning electron microscopy (SEM) was used to investigate the morphologies of the polypyrrole nanotubes; the images shown herein are representative, and at least seven points from different syntheses were analyzed to ensure both representativity and reliability. Spectroscopic characterization of the materials after adsorption was performed using infrared spectroscopy, and the spectra of the modified electrodes were taken directly on the electrode in transmittance or attenuated total reflectance (ATR) mode with a germanium crystal attachment. The analysis was performed on the solid sample without any additional preparation.

### 2.4 Electrochemical Measurement

To investigate the electrochemical performance of the as-prepared electrodes, cyclic voltammetry (CV) was used. The tests were measured in a three-electrode system using the prepared electrode as a working electrode, Ag/AgCl/Cl^–^(sat) as a reference electrode, a large-area platinum spiral as a counter electrode, and a 0.1 mol L^–1^ KCl solution as an electrolyte solution. The CV curves were obtained in a potential range from −0.8 up to 0.5 V at scan rates of 1, 5, 10, 20, and 50 mV s^–1^. Electrochemical impedance spectroscopy (EIS) was performed in open-circuit potential (OCP) superimposed by an *ac* potential of 0.01 V in a frequency range from 10 kHz to 10 mHz. To ensure that the EIS measurement was performed under steady-state conditions, the working electrode was polarized at the *dc* potential until the stabilization of the minimum current values. ZView software was used to fit the EIS results.

### 2.5 Electrosorption or Adsorption with Potential Control

Caffeine adsorption was carried out in an electrochemical cell using 0.1 mol L^–1^ KCl as an electrolyte enriched with different concentrations of caffeine (2, 5, 10, 15, and 30 mg L^–1^) under magnetic stirring at 150 rpm. The modified electrode was then immersed in the electrolyte (with and without caffeine); the potential application was kept constant until the adsorption reached equilibrium. Caffeine adsorption was monitored by a UV–vis spectrophotometer (Model: Varian Cary60 from Agilent Technologies) at a wavelength of 273 nm, and a proper calibration curve was used to reach the concentration of the samples.

The quantity of adsorbate uptake by electrodes was calculated by a mass balance relationship, which represents the amount of adsorbed compound per amount of adsorbent using eq 1:

1 $$q_{e}=\frac{\left(C_{0}-C_{e}\right)}{m}\times V$$

where *C*_0_ is the initial caffeine concentration [mg L^–1^], *C*_e_ is the caffeine concentration after adsorption [mg L^–1^], *V* is the volume of the caffeine solution [L], and m is the electrode mass [g].^31^

### 2.6 Adsorption Isotherms

Adsorption isotherms were constructed at room temperature. To construct the isotherms, adsorption tests were carried out using a potential of −0.8 V (vs Ag/AgCl/Cl^–^(sat)) until the equilibrium was reached, with variations in the caffeine concentration in the solution of 1, 2, 5, 10, 15, and 30 mg L^–1^. The Sips model was used to fit the experimental results;^32–34^ the equation is shown below ([Disp-formula eq2]):

2 $$q_{e}=\frac{q_{ms}K_{s}C_{e}^{n_{s}}}{1+K_{s}C_{e}^{n_{s}}}$$

where *q*_ms_ is the adsorbed amount of a monolayer (mg g^–1^) and *K*_s_ (L^*n*^_s_mg^–*n*^_s_) and n_s_ are the Sips constants. It is important to mention that the Sips model is a combination of the Langmuir and Freundlich models, becoming the Langmuir model when n_s_ assumes the unity value and the Freundlich model at low *C*_e_ values ( $1\gg K_{s}C_{e}^{n_{s}}$ ).^35^

## 3 Results and Discussion

### 3.1 Morphological and Electrochemical Results

The morphologies of the modified electrodes were verified by SEM images, as can be seen in Figure 1. The overall morphology of the PPyNTs consists of distributed nanotubes along the steel mesh electrode with a few micrometers in length and a few hundreds of nanometers in diameter, corroborating the works published elsewhere.^28^ The electrodeposition of SiO_2_ occurred directly on the surface of the conducting nanotubes, creating a cloudy structure with no specific shape, but clearly those structures are electrically connected with the PPyNTs. This observation contrasts with the highly ordered mesoporous silicas, with channels oriented perpendicular to the substrate, obtained by Electro-Assisted Self-Assembly (EASA) routes described by Goux et al.^36^ and Walcarius et al.^37^ The formation of particulate aggregates with a small particle size in our system may be influenced by the presence of MO residues, used in the synthesis of PPyNTs. The MO could have acted by altering the nucleation and growth of the silica and promoting disordered aggregation. This occurrence of particulate aggregates on the surface is a phenomenon reported by Goux et al.^36^ and Walcarius et al.^37^ for thicker EASA films, where bulk gelation at the electrode/solution interface, out of the control of directed self-organization, leads to the formation of byproducts with poorly defined structure. However, to confirm this, a more in-depth study is necessary. The presence of silica in the electrodes was also confirmed by the EDS mapping technique (Figure S1). The PPyNTs/SiO_2_ electrode presented a large amount of silicon in the sample compared to the electrode with only PPyNTs, indicating the formation of SiO_2_ well distributed over the PPyNT-modified electrode surface.

**Figure 1:**
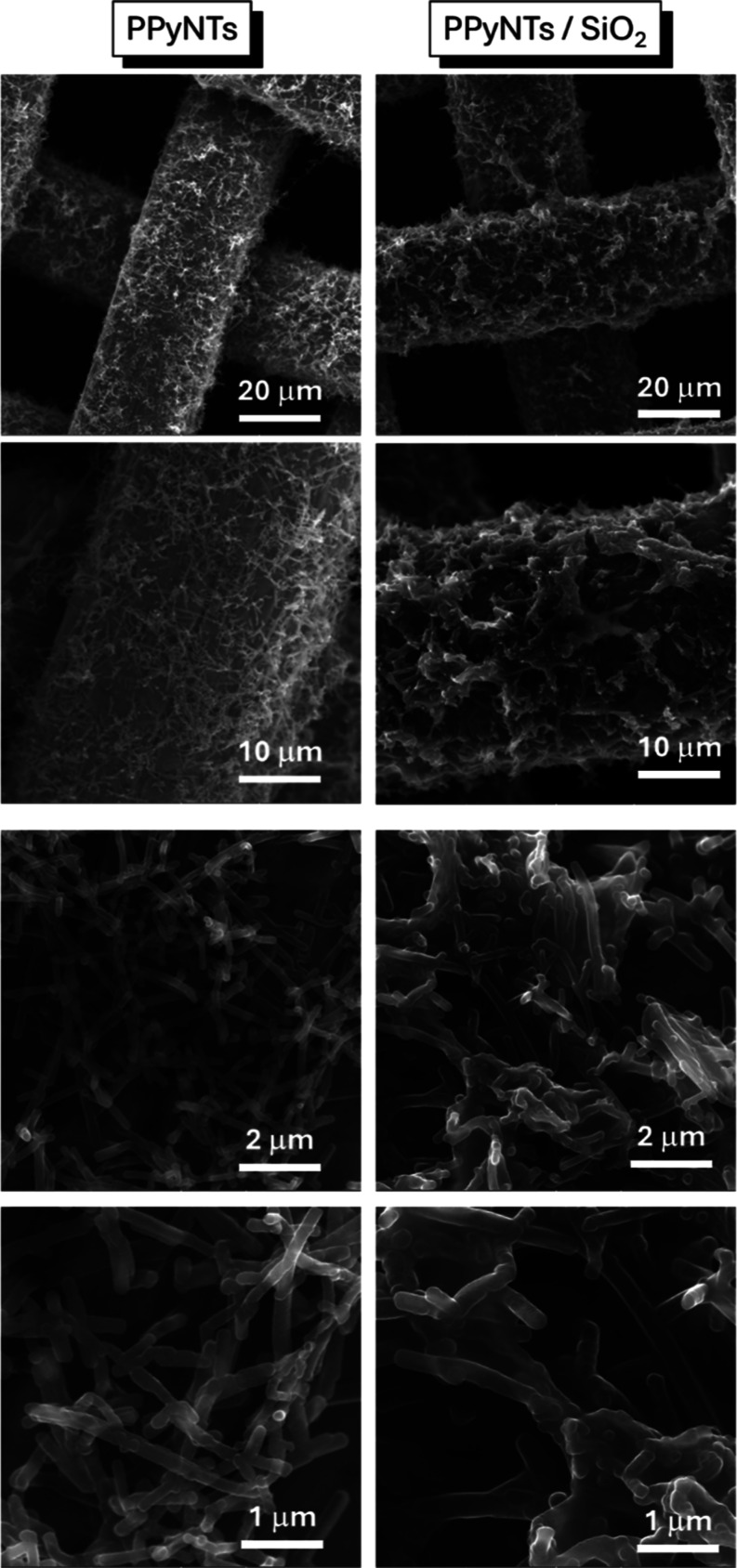
Representative SEM images from modified electrodes of PPyNTs and PPyNTs/SiO_2_.

The modified electrodes were characterized by cyclic voltammetry in aqueous 0.1 mol L^–1^ KCl (Figure 2(a)). In the black line, the PPyNTs show the typical response of large-area electroactive surfaces, presenting an extensive capacitive behavior with the polypyrrole reversible redox peaks.^38^ In the presence of SiO_2_, the red line, the oxidation peak of PPyNTs shifts toward higher energy, indicating a slower oxidation process of PPy; in the reverse scan, there is no drastic change in the format or the reduction peak; as described in literature,^39^ the less oxidized form of PPy has lower conductivity if compared to the partially and fully oxidized states, which explains the overpotential needed to oxidize the PPyNTs in the presence of SiO_2_ (a semiconductor material with high Eg 4.9 eV^40^), with no drastic change in the reversible reduction of the material.

**Figure 2:**
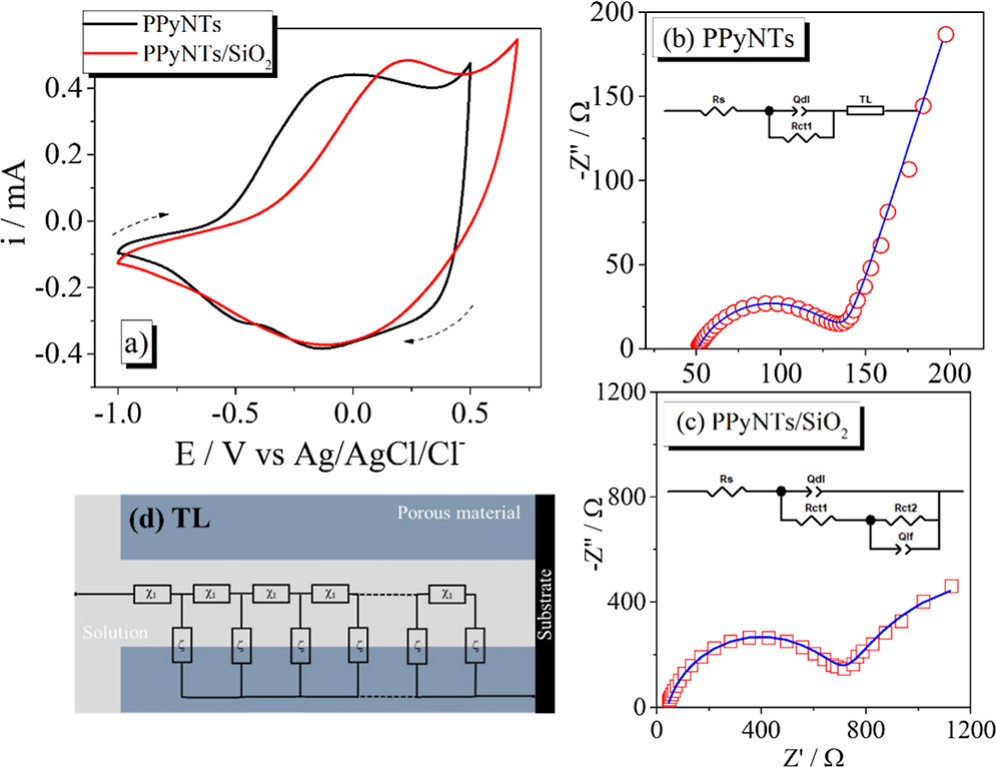
(a) CV curves of PPyNTs and PPyNTs/SiO_2_ electrodes in 0.1 mol L^–1^ KCl aqueous solution at 20 mV s^–1^. Nyquist plots of (b) PPyNTs and (c) PPyNTs/SiO_2_-modified electrodes and (d) general representation of a transmission line equivalent circuit. EIS measurements were performed in open-circuit potential (OCP) superimposed by an *ac* potential of 0.01 V in a frequency range from 10 kHz to 10 mHz.

The modified electrodes were also characterized by EIS in open-circuit potential (OCP) superimposed by an *ac* potential of 0.01 V in a frequency range from 10 kHz to 10 mHz, and the results are shown in Figures 2b and 2c for PPyNTs and PPyNTs/SiO_2_, respectively. The Nyquist plot of the PPyNTs presents a typical response of a conducting polymer with a semicircle in the high and medium frequencies corresponding to the charging of the double layer and the charge transfer at the electrode/electrolyte interface, respectively. A straight line is found at lower frequencies, associated with the intercalation of ionic species in the polymeric matrix to maintain charge neutrality during the redox reactions.^41^ Though, a closer look into the EIS response makes it possible to identify a straight line approaching 45° in the high-frequency region, as depicted in the spectrum (this region is detailed in Figure S2). This characteristic is indicative of the highly porous nature of the PPyNT morphology. To better extract the parameters of this material, a proper equivalent circuit methodology can be addressed, composed of two closely mixed phases presenting a certain degree of disorder in the electroactive material, with narrow pathways for simultaneous transport of ionic and electronic species in the liquid and solid phases, respectively.^42^ Taking that into account, a transmission line (TL) methodology was considered herein as described in ref 43; the respective circuit is inserted in Figure 2b.

The proposed equivalent model considers the intrinsic conductivity of the poly­(pyrrole), so it can be assumed that the charge transport is performed by the ionic species throughout the whole porous morphology. In this way, a single-channel TL was considered to represent the ionic transport inside the pore (χ_l_), as well as the intercalation of such ionic species in the polymeric matrix to maintain charge neutrality (ξ), as shown in Figure 2d. The usual restriction was adopted that the quantities χ_l_ and ξ are adequately described by unique functions of frequency, independent of position in the layer; in addition, the quantities χ_l_ and ξ were represented by a resistance (*r*_pore_) and a constant phase element (*q*_lf_).^42,44^ The other parameters represent the series resistance of the system, including the electrolyte, wires. and connections (*R*_s_); the resistance of the charge transfer at the electrode/electrolyte interface (*R*_ct_); and a constant phase element (*Q*_dl_) accounting for the double-layer capacitance.^41^ All results were fitted using the software ZView to determine the EIS parameters. The results are shown in Table 1, to be discussed ahead, just after the spike of caffeine in the electrolyte.

**Table 1:** Quantitative Results Obtained from the Modeling of the Nyquist Plots Obtained in Figure 2b,c and Figure 3c,d^a^

sample	*R*_s_/Ω	*Q*_dl_/10^–4^ F.s^*n*–1^	*n*_dl_	*R*_ct1_/ Ω	*r*_pore_/Ω	*q*_lf_/10^–2^ F.s^*n*–1^	*n*_lf_	*Q*_lf_/ 10^–2^ F.s^*n*–1^	*n*_lf_	*R*_ct2_/Ω
**PPyNT**										
**0 ppm**	50.52	1.99	0.73	75.31	34.02	1.58	0.81	-	-	-
**15 ppm**	46.77	0.81	0.85	196.1	71.74	1.36	0.78	-	-	-
**30 ppm**	49.84	0.54	0.88	734.1	252.8	1.24	0.88	-	-	-
**PPyNT/SiO**_**2**_										
**0 ppm**	40.55	2.10	0.82	703.6	-	-	-	1.28	0.83	1208
**15 ppm**	38.87	1.05	0.86	1438	-	-	-	1.10	0.94	1263
**30 ppm**	37.74	0.77	0.87	2131	-	-	-	0.75	0.77	3514

a Equivalent circuit methodology employed, *R* > 0.99.

The incorporation of SiO_2_ in the PPyNTs matrix provoked some changes in the Nyquist plot, as shown in Figure 2c. The TL pattern is no longer observed; instead, a second semicircle appeared in the low-frequency region, which indicates a second charge transfer kinetics, probably at the SiO_2_ interface.^9^ Also, according to the SEM images, clearly the SiO_2_ blocks the interface of PPyNTs and the overall porosity of the modified electrode. Considering these modifications, another equivalent circuit is proposed, consisting of a resistance regarding the second charge-transfer process (*R*_ct2_) in parallel to a constant phase element (*Q*_lf_) accounting for the intercalation process in the polymeric matrix to maintain charge neutrality. The circuit is inset in Figure 2(c). The calculated values using the respective equivalent circuit model presented in Figure 2 are shown in Table 1.

In the presence of caffeine, neither modified electrode showed any signal of faradaic response, as observed in Figures 3a and 3b, indicating that there is no electrocatalytical behavior of PPyNTs or PPyNTs/SiO_2_-modified electrodes. Within the range of concentration tested, the PPyNT electrode showed no drastic change in the voltammetric behavior; a slight decrease in the current was found, probably due to the adsorption of caffeine at the interface. On the other hand, in the presence of SiO_2_, the effect of the adsorbed caffeine is highly pronounced with the decrease of the current according to the caffeine concentration, suggesting a strong fouling effect, blocking the electroactive sites of the material. These adsorption effects were also tested using EIS as observed in Figures 3c and 3d.

**Figure 3:**
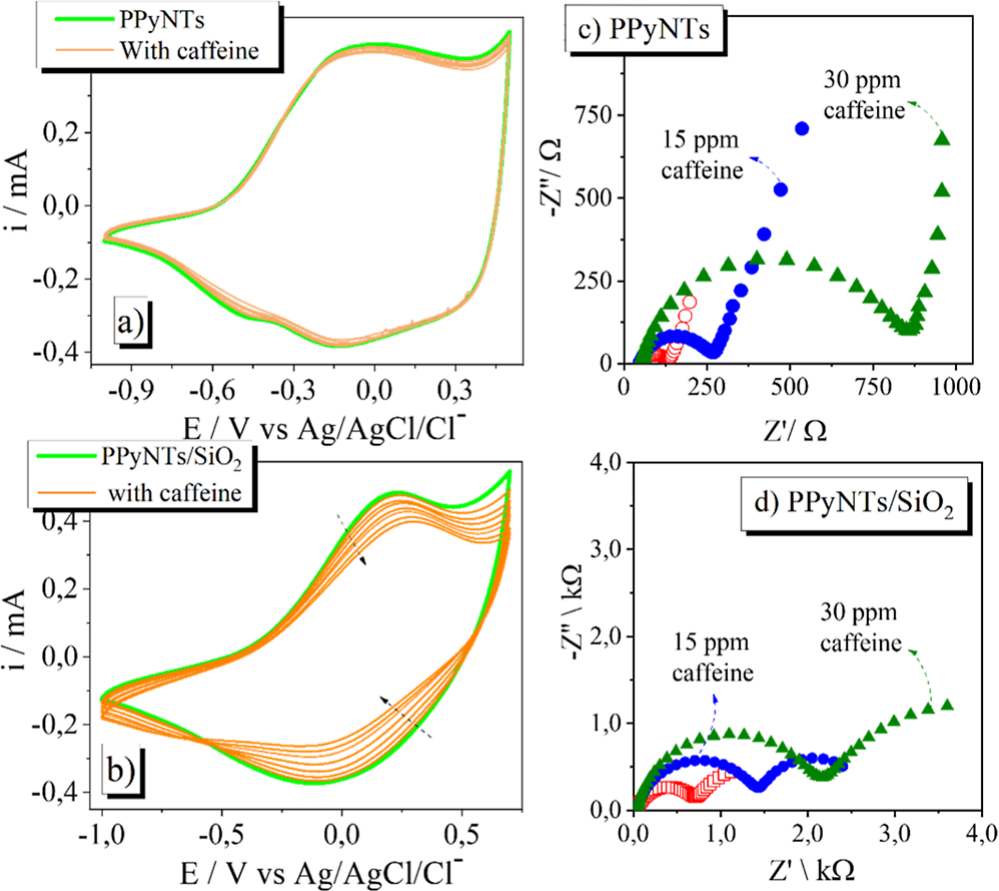
(a) CV curves of PPyNTs and (b) PPyNTs/SiO_2_ electrodes in 0.1 mol L^–1^ KCl aqueous solution spiked with different concentrations of caffeine from 1 up to 30 ppm, with a scan rate of 20 mV s^–1^. Nyquist diagrams of (c) PPyNTs and (d) Nyquist diagrams of PPyNTs/SiO_2_ in different concentrations of caffeine. EIS measurements were performed in open-circuit potential (OCP) superimposed by an *ac* potential of 0.01 V in a frequency range from 10 kHz to 10 mHz.

In both modified electrodes, the presence of caffeine has changed the Nyquist plots similarly, increasing the semicircle diameter, which indicates an increase in the charge transfer resistance (*R*_ct_ and *R*_ct2_) at the electrode interface. As commented earlier, this corroborates the adsorption of caffeine at the PPyNT interface and most strongly in the silica-modified electrodes, where the blocking is more intense, observing the *R*_ct_ values found in Table 1. In the same perspective, the increase in the *r*_pore_ values is also attributed to the caffeine adsorption, followed by the diminishment of the capacitance of the double layer, as seen in the *Q*_dl_ values, and by the increase in the *n*_dl_ values, indicating more homogeneity at the surface. The diminishment of the *Q*_lf_ (*q*_lf_) values can be attributed to the decrease of the loading of intercalated ions in the polymeric matrix to maintain charge neutrality, which is consistent with the loss of electroactivity of the conducting polymer due to fouling effects.

### 3.2 Electrosorption of Caffeine, Mechanistic Studies

The adsorption capacity of caffeine under different electrochemical potentials was done according to the scheme shown in Figure 4a; the caffeine concentration in the electrolyte can be easily obtained by monitoring the band at 273 nm, and the respective concentration can be found.^9^ Figure 4b shows the adsorption capacity at different applied potentials, and the experiment was done in triplicate. It is possible to verify that at more negative potential there is a drastic increase in the caffeine adsorption at the modified electrodes; this effect is related to the presence of less oxidized PPy structure, as the presence of the π-π localized orbitals interacts to a greater extent with organic molecules.^45^

**Figure 4:**
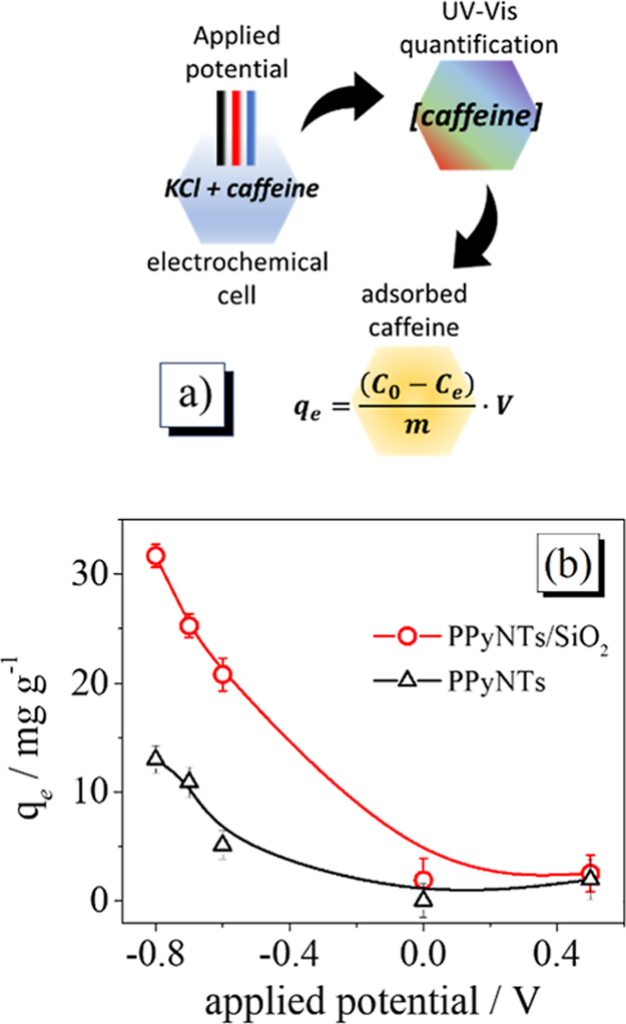
(a) Schematic representation of q_e_ parameter calculus; (b) 30 ppm of caffeine adsorption capacity of the PPyNTs/SiO_2_ composite electrode at different voltages in 0.1 mol L^–1^ KCl.

To obtain information about the mechanism of the adsorption process and the interaction of PPyNTs and PPyNTs/SiO_2_ electrodes with caffeine, the adsorption isotherms were analyzed by the Sips mathematical model,^46^ as commented earlier. It is important to mention that Langmuir and Freundlich models were also tested, though with lower accuracy of the data obtained. The results are shown in Figure 5 and Table 2.

**Figure 5:**
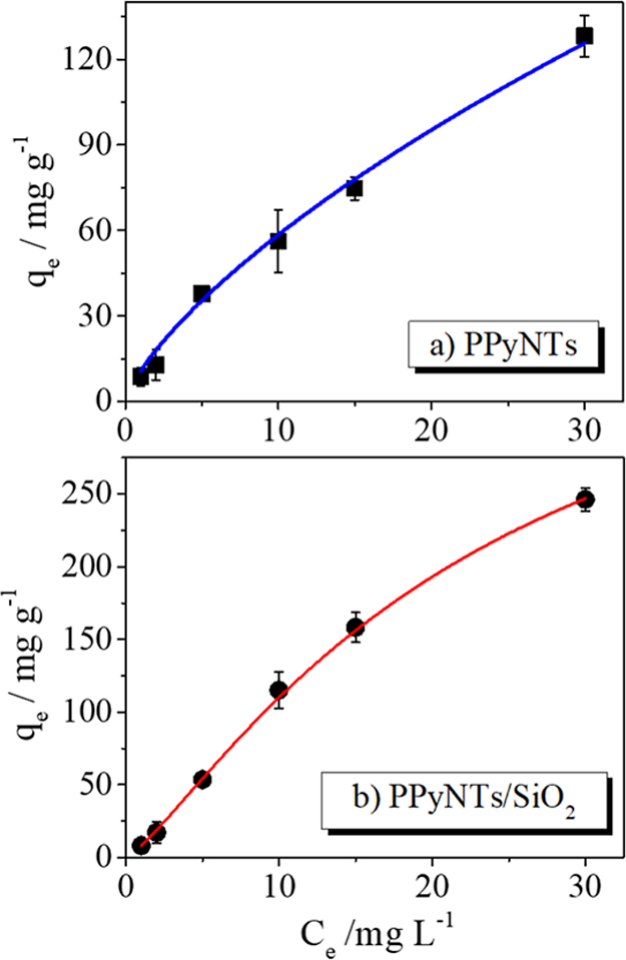
Sips modeling of the experimental adsorption results using (a) PPyNTs and (b) PPyNTs/SiO_2_-modified electrodes. Electrolyte: 0.1 mol L^–1^ KCl. Experiments in triplicate.

**Table 2:** Data Obtained from Sips Mathematical Fitting Isotherms for PPyNTs and PPyNTs/SiO_2_ Electrodes

	PPyNTs	PPyNTs/SiO_2_
*q*_ms_	0.009 ± 0.001	0.0190 ± 0.0009
*K*_s_	11 ± 2	8.3 ± 0.2
*n*_s_	0.75 ± 0.03	1.25 ± 0.02
*R* ^2^	0.99	0.99

The Sips isotherm is a combination of the Langmuir and Freundlich models, developed to predict heterogeneous adsorption systems and to overcome the limitation of the adsorbate concentrations. The Sips model indicates that the PPyNTs/SiO_2_ electrode has a greater affinity for caffeine in addition to being a more heterogeneous material than the electrode with only PPyNTs, corroborating what was observed in the SEM images.

It is observed through the Sips model parameters that the PPyNTs/SiO_2_ electrode presented a significantly higher *q*_ms_ value (0.019) compared with the pure PPyNT electrode (0.009). The *q*_ms_ parameter represents the maximum amount of adsorbate that can be adsorbed on the surface under ideal conditions, reflecting the total adsorption capacity of the material.^34^ The increase in *q*_ms_ for PPyNTs/SiO_2_ clearly indicates a higher caffeine adsorption capacity. This result suggests that the incorporation of silica not only maintains the adsorption capacity of PPyNTs but also enhances it, possibly by increasing the accessible surface area or creating new adsorption sites. The *K*_S_ parameter provides information about the affinity between the adsorbate and the adsorption sites.^47^ It is observed that PPyNTs/SiO_2_ has a slightly lower *K*_S_ value (8.3) compared to that of PPyNTs (11). Although a lower *K*_S_ may, at first glance, suggest a slightly reduced affinity for individual sites, the interpretation should be made in conjunction with the *n*_S_ parameter. The heterogeneity exponent *n*_S_ is a parameter that reflects the degree of heterogeneity of the adsorption surface. *n*_S_ values close to 1 indicate a more homogeneous surface (approaching the Langmuir model), while values greater or less than 1 suggest heterogeneity.^8,34,47^ The pure PPyNT electrode presented a *n*_S_ of 0.75, indicating some heterogeneity on its surface. In contrast, the PPyNTs/SiO_2_ electrode exhibits a notably higher *n*_S_ (1.25). This increase in *n*_S_ for PPyNTs/SiO_2_ strongly corroborates the observations from the SEM images. This complex and irregular morphology of silica introduces a variety of adsorption sites with different energies and accessibilities, confirming that the hybrid material has an intrinsically more heterogeneous surface. Heterogeneity, in this case, is beneficial because it allows caffeine to interact in multiple ways with the surface, through functional groups of PPyNTs and silica (such as silanol groups), or through different site geometries. Therefore, the combination of a higher maximum adsorption capacity (*q*_ms_) with a higher surface heterogeneity (*n*_S_) makes the PPyNTs/SiO_2_ electrode more effective for caffeine adsorption. In summary, the incorporation of silica into PPyNTs promoted the formation of a highly heterogeneous surface, which, together with the increase in surface area and the introduction of new interaction sites, resulted in a significantly improved caffeine adsorption capacity.

### 3.3 Structural Characterization and Caffeine Effects

The modified electrodes were characterized before and after caffeine exposure; the results are shown in Figure 6. It is not the scope of this work to provide a full description of the spectroscopic features of PPyNTs according to the literature,^48^ but the effects caused by the incorporation of SiO_2_ and the changes in the presence of caffeine and the pristine spectra of SiO_2_ and caffeine are shown in Supporting Information S2. The presence of electrodeposited SiO_2_ structures has not affected the overall spectrum of PPyNTs/SiO_2_ apart from a slight enlargement of the band centered in 1177 cm^–1^, corresponding to the ring deformation of poly­(pyrrole);^49^ this band is very symmetric in the PPyNT spectrum but overtones with the most intense band region of SiO_2_, in particular with the bands at 1190 and 1094 cm^–1^, related to asymmetric stretching vibrations of the Si–O–Si bridging bonds of SiO_2_.^50^

**Figure 6:**
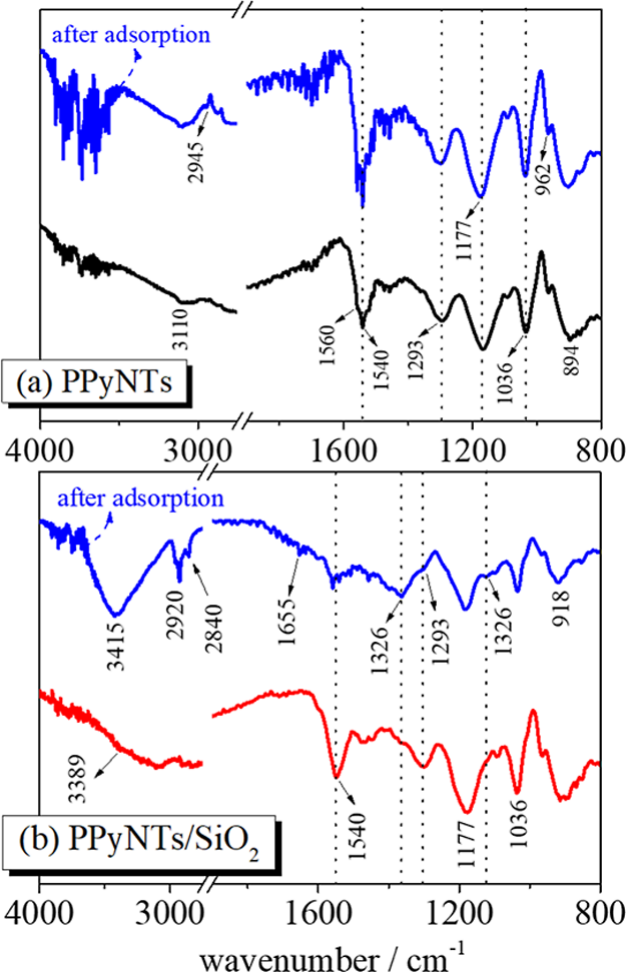
(a) Analytical curve in 0.1 mol L^–1^ KCl; (b) 30 ppm of caffeine adsorption capacity of the PPyNTs/SiO_2_ composite electrode at different voltages in 0.1 mol L^–1^ KCl.

In the absence of SiO_2_, Figure 6(a), the pristine PPyNTs show discrete changes comparing the modified electrode before and after caffeine exposure. Any band of PPyNTs has presented a strong modification that would suggest a strong interaction of caffeine; neither of their strongest bands are present in the spectra, but its presence could be noticed by the noisy signal in higher frequencies and in the region around 1600–1800 cm^–1^ (this experiment was repeated three times, and this behavior is very reproducible), which would also suggest a very weak interaction with the interface of PPyNTs. On the other hand, the PPyNTs/SiO_2_-modified electrode has some important modifications; the presence of caffeine can be observed by the strong bands at 3415, 2920, and 2840 cm^–1^; compared to the pure spectrum of caffeine, these bands were found at 3450, 3114, and 2948 cm^–1^, corresponding to the vibrational modes of –CH_3_ groups attached to the nitrogen. This behavior indicates the strong interaction with the PPyNTs/SiO_2_ surface; this same behavior was found in a work by Danish and coauthors studying the adsorption of caffeine in activated carbon,^51^ one of the strongest adsorbent materials found. Also, some bands of PPyNTs/SiO_2_ had suffered a drastic diminishment of relative intensity, such as 1540 and 1293 cm^–1^, corresponding to the standard stretching mode of the pyrrole ring (C=C) and C–N stretching vibration of the pyrrole ring, respectively,^52^ indicating that these groups interact greatly with caffeine during the adsorption. As commented during the electrosorption, the presence of C=C bonds is more suitable for the interaction with caffeine, which also increases with the SiO_2_, suggesting a synergistic effect between these two structures.

The proposed adsorption mechanism involves π–π interactions with PPyNTs and the formation of hydrogen bonds between the caffeine carbonyl group (C=O) and the silica silanol groups (Si–OH). Since these interaction sites are not exclusive to caffeine, other molecules with similar structural motifs might act as interferents. Pharmaceuticals (e.g., paracetamol, ibuprofen, and naproxen), other xanthine derivatives (e.g., theophylline), and natural organic matter (e.g., humic and fulvic acids) possess aromatic rings or functional groups capable of competing for the same active sites, potentially affecting caffeine adsorption. Although this study establishes a proof-of-concept for the electrochemically controlled adsorption of caffeine, the quantitative evaluation of the interferents is beyond its scope.

### 3.4 Desorption

After the adsorption process to concentrate the analyte on the electrode surface or on any other sorption material, the desorption step is necessary to remove the analyte from the electrode for later determination or to regenerate this material. In electrosorption, electrode regeneration is usually performed by short-circuiting or applying a reverse voltage to desorb electroadsorbed ions from the electrode.^53^ In this work, the desorption process was tested by applying reverse potential and monitored by the UV–vis technique, following the same adsorption procedure described in item 2.5. The results obtained are shown in Figure 7, and it can be noted that all electrodes tested and at all concentrations tested entered desorption equilibrium in approximately 40 min. The PPyNTs/SiO_2_ electrodes presented a lower desorption capacity than the PPyNT electrodes, possibly due to the presence of two adsorption materials, indicating that silica interacts strongly with caffeine, hindering the regeneration process. The maximum desorption achieved was approximately 27% for the PPyNT electrode at a concentration of 15 ppm. These results show that the developed material may be interacting strongly with caffeine, which hinders the desorption process. Studies using solvents that are normally used in the regeneration of adsorptive materials were not performed, as this was not the focus of the work at the moment.

**Figure 7:**
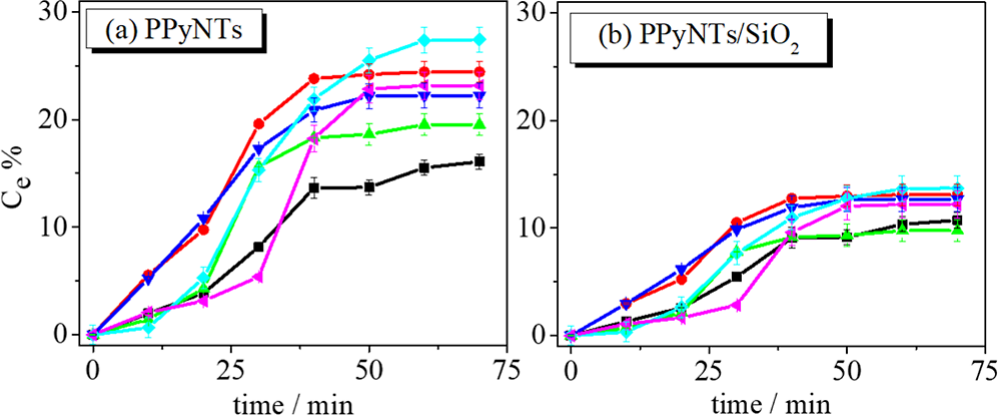
Desorption capacity of (b) PPyNTs/SiO_2_ electrodes and (a) PPyNTs with an applied potential of 0.8 V in 0.1 mol L^–1^ KCl.

## 4 Conclusion

The electrosynthesis of PPyNTs/SiO_2_-modified electrodes presented an easy and rapid methodology for the construction of organic–inorganic hybrid materials; in particular, this modified electrode has shown a high caffeine sorption effect, tunable by electrochemical control. The highest sorption capacity at the potential at −0.8 V (vs Ag/AgCl/Cl^–^(sat), mainly due to the decrease of positive charges in the lowest oxidation state of polypyrrole, enhances the π–π interactions and the overall adsorption feature of SiO_2_. EIS experiments have supported the Sips adsorption isotherm and showed a powerful technique to further characterize sorption phenomena. This work presents a new perspective for future analytical advances for caffeine detection in aqueous samples by combining high adsorption efficiency with electrochemical control.

## Supplementary Material



## Notes

EDS mapping of the PPyNTs and PPyNTs/SiO_2_-modified electrodes and detailed region of the EIS spectrum at higher frequencies (PDF)
