# Supplementary material for: Interfacial Characterization of the Electrochemical Adsorption of Caffeine on Poly(pyrrole) Nanotubes/Silica
Source: ACS Omega. 2025 Nov 21;10(48):59895–903. doi: 10.1021/acsomega.5c10006 (PMC12772410; doi:10.1021/acsomega.5c10006)

Interfacial characterization of the electrochemical adsorption of caffeine on poly(pyrrole) nanotubes / silica

Tatiana Lima Valerio[A], Camilla K. Boaron[A], Luis F. Marchesi,[A,B] Bruno José G. da Silva[A,C], Marcio Vidotti[A]*

A - Grupo de Pesquisa Em Macromoléculas e Interfaces (GPMIn), Departamento de Química, Universidade Federal Do Paraná (UFPR), CP 19032, 81531-980, Curitiba, PR, Brazil

B - Grupo de Estudos em Espectroscopia de Impedância Eletroquímica (GEIS), Universidade Tecnológica Federal Do Parana, Rua Dr.Washington Subtil Chueire, 330. Jd. Carvalho, CEP 84017-220, Ponta Grossa, PR, Brazil

C - Grupo de Cromatografia e Técnicas de Microextração (CROME) - Departamento de Química, Universidade Federal do Paraná, C.P. 19032, 81531-980, Curitiba, PR, Brazil

Figure S1. Chemical mapping for the elements carbon, nitrogen, oxygen and silicon in (a) PPyNTs/SiO2 and (b) PPyNTs electrodes

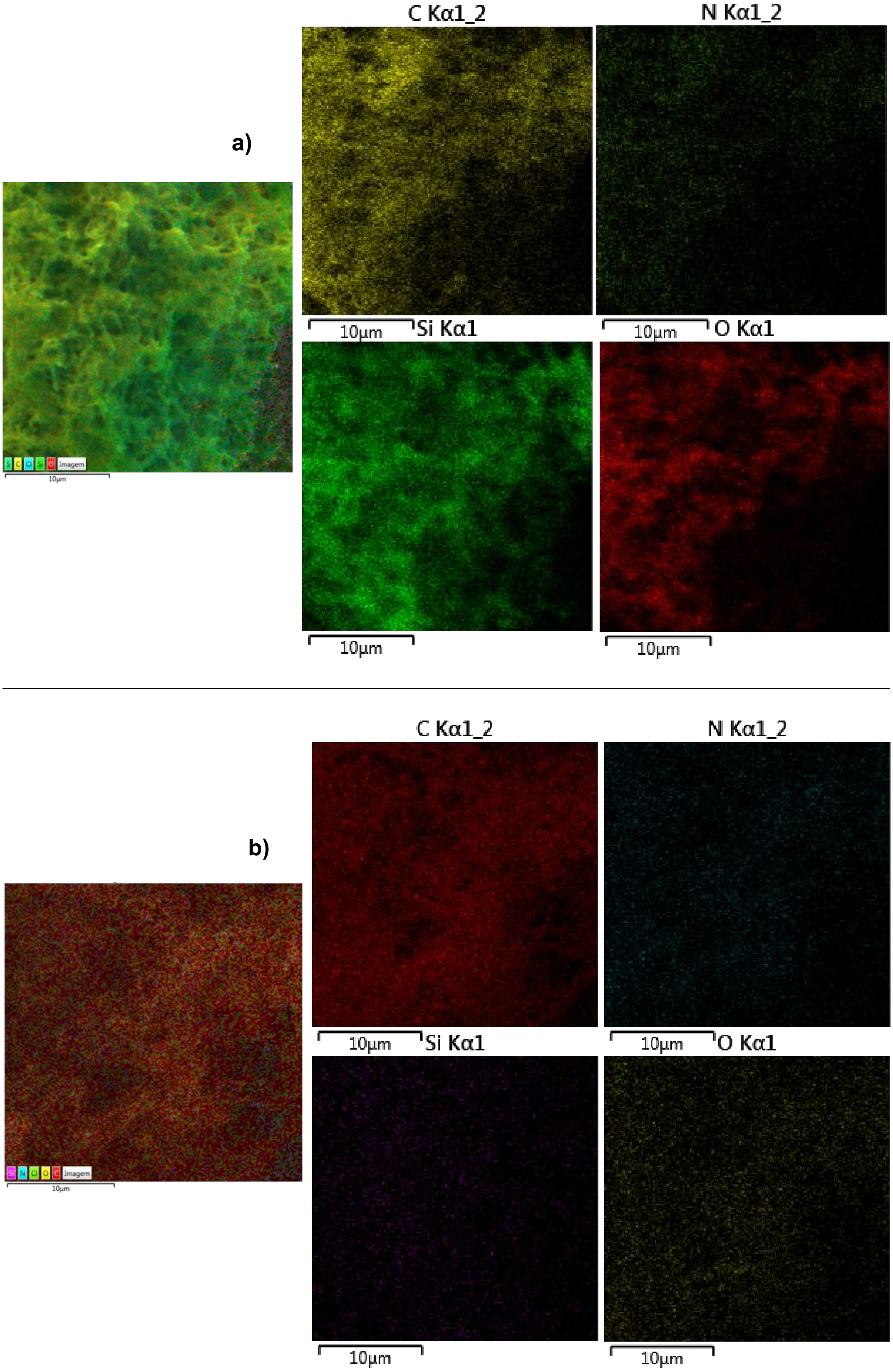

Figure S2. Nyquist plot presented in Figure 2b, showing the 45º region at higher frequencies of the EIS result.

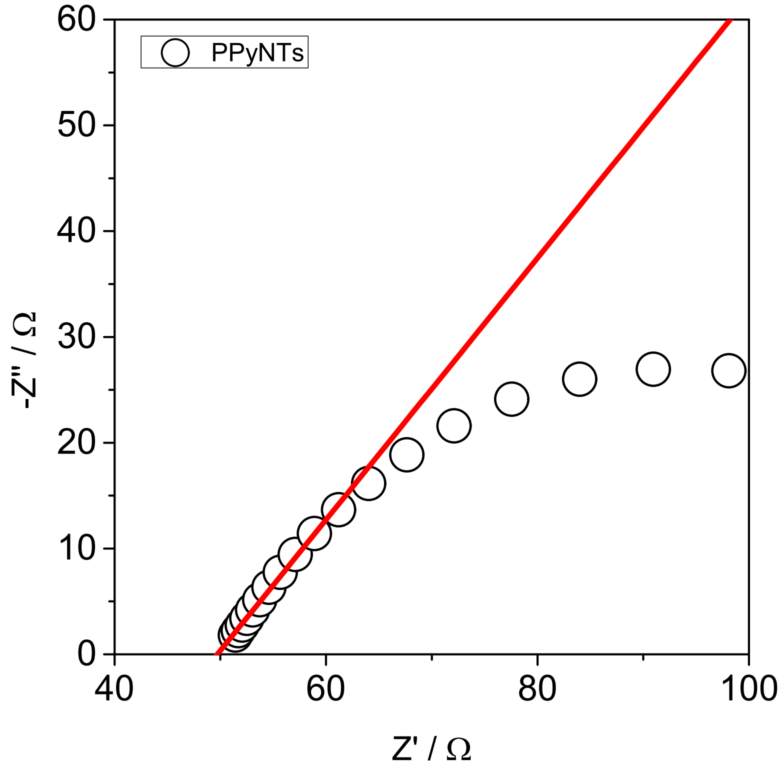

Supplement: Supplementary file 1 [file ao5c10006_si_001.pdf]
